# Modified radical mastectomy sparing one or both pectoral muscles in the treatment of breast cancer: intra and postoperative complications

**DOI:** 10.1590/S1516-31802006000300004

**Published:** 2006-05-04

**Authors:** Ruffo Freitas, Evelling Lorena Cerqueira Oliveira, Rubens José Pereira, Marco Aurélio Costa Silva, Maurício Duarte Esperidião, Rossana Araújo Catão Zampronha, Luiz Fernando Jubé Ribeiro, Geraldo Silva Queiroz, Estanislau Araújo Jorge, Rosemar Macedo Sousa Rahal, Júlio Eduardo Ferro, Régis Resende Paulinelli, Silvânia Fátima Coelho Barbosa

**Keywords:** Breast cancer, Breast, Mastectomy, Techniques, Complications, Neoplasms, Câncer de mama, Mama, Mastectomia, Técnicas, Tratamento, Complicações, Neopla**sias**

## Abstract

**CONTEXT AND OBJECTIVE:**

Modified radical mastectomy is widely utilized in breast cancer treatment. However, no prospective comparison has yet been made between the Madden technique (preservation of the pectoralis minor muscle) and the Patey technique (resection of this muscle). The aim of this work was to compare these two modified radical mastectomy techniques, by analyzing their degrees of difficulty and complications.

**DESIGN AND SETTING:**

Randomized trial at the Breast Unit of Hospital Araújo Jorge, Goiás; and Faculdade de Medicina da Universidade Federal de Goiás.

**METHODS:**

430 patients with breast cancer with an indication for modified radical mastectomy were included in the program, of whom 426 patients were available for analysis (225 allocated to Patey and 201 to Madden). The chi-squared and Student t tests were used for analysis.

**RESULTS:**

The patients’ demographics were well balanced between the two groups. The mean duration of the surgical procedures was 105 (± 29.9) and 102 minutes (± 33), for the Patey and Madden groups, respectively (p = 0.6). Hospitalization duration was 2.3 days for both groups. The mean number of lymph nodes resected was 20.3 (± 7.6) for Patey and 19.8 (± 8.1) for Madden (p = 0.5). There were no differences in terms of vascular or nerve sections, hematomas or infections. The surgeons reported the same degree of difficulty for the two methods.

**CONCLUSION:**

The removal of the pectoralis minor muscle did not influence any of the variables studied. Therefore, either technique can be performed, at the surgeon's discretion.

## RESUMO

**CONTEXTO E OBJETIVO:**

A mastectomia radical modificada continua a ser amplamente usada para o tratamento de câncer de mama. Porém, até agora, a preservação do músculo peitoral secundário (técnica de Madden) não foi prospectivamente comparada à técnica de Patey. O objetivo deste trabalho foi comparar as duas técnicas de mastectomias radicais modificadas, analisando o grau de dificuldade e as complicações.

**TIPO DE ESTUDO E LOCAL:**

Estudo randomizado, realizado na Unidade de Mama do Hospital Araújo Jorge, Faculdade de Medicina da Universidade Federal de Goiás, Goiás, Brazil.

**MÉTODOS:**

430 pacientes portadoras de câncer de mama com indicação de mastectomia radical modificada foram incluídas no programa. Foram disponíveis para análise 426 pacientes, das quais 225 alocadas no grupo Patey e 201 no grupo Madden. A análise foi feita por intenção de tratamento, usando-se o Qui-quadrado ou o teste t de Student, quando aplicáveis.

**RESULTADOS:**

A distribuição das características demográficas pacientes foi semelhante entre os grupos. A duração média da cirurgia foi de 105 minutos (DP ± 29.9) e 102 (DP ± 33) para o grupo Patey e Madden, respectivamente (p = 0,6). O tempo de internação foi de 2,3 dias para ambos os grupos. A média de linfonodos ressecados foi de 20,3 (DP ± 7,6) para Patey e 19,8 (DP ± 8,1) para Madden (p = 0,5). Não houve diferenças entre as complicações vasculares, nervosas, hematomas, infecções, bem quanto à dificuldade relatada pelo cirurgião.

**CONCLUSÃO:**

A retirada do músculo pequeno peitoral não influenciou nenhuma das variáveis estudadas. As técnicas de mastectomias radicais modificadas, Patey e Madden, foram semelhantes em todos os critérios observados, podendo ser executadas de acordo com a preferência do cirurgião.

## INTRODUCTION

In spite of great advances in surgery, especially with regard to breast-conserving therapy, which has already been proven to have long-term efficacy,^1,2^ modified radical mastectomy is still widely used around the world. Especially in developing countries, there are significant numbers of cases in which the tumor has already reached an average size of 4 cm upon diagnosis. This results in thousands of women having to undergo modified radical mastectomy every year.^3–5^

In view of the lack of studies comparing the two techniques for modified radical mastectomy, i.e. sparing one or both pectoral muscles, we endeavored to develop this randomized trial.

## OBJECTIVE

To identify which of the two modified radical mastectomy techniques (Patey or Madden^6,7^) could result in a smaller number of intra and postoperative complications, and also to determine the number of lymph nodes that would be resected while performing axillary clearance.

## PACIENTS and METHODS

This randomized trial compared two modified radical mastectomy groups: sparing the pectoralis major muscle (Patey) or sparing both pectoralis muscles (Madden). The trial included 430 patients from the Breast Unit of Hospital Araújo Jorge, Goiás Anticancer Association, and from the Department of Gynecology and Obstetrics of Universidade Federal de Goiás, with an indication for modified radical mastectomy for operable breast cancer, between April 1997 and April 2002. The trial did not include patients who had undergone radiotherapy before the surgery; patients with previous concomitant neoplasia other than cases of adequately treated cervical intraepithelial neoplasia (CIN III) or skin cancer; diabetic patients; or patients with an indication for classical radical mastectomy.

The study protocol was granted prior approval by the Ethics Committees of both institutions. Once the patients agreed to take part in the study, through signing the informed consent form, they were randomly allocated to the Patey or Madden groups, according to the surgical technique that was going to be used. The randomization process consisted of the allocation of patients according to a central logbook numbered from 1 to 430, distributed randomly to either group.

**Figure 1:**
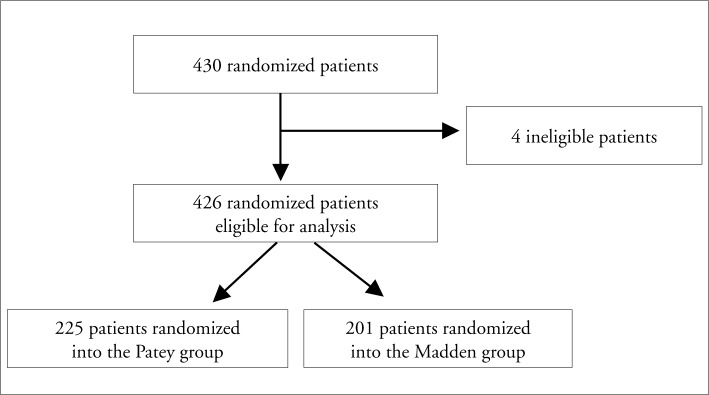
Flow diagram summarizing how patients were allocated in the study. Patey group included women who underwent mastectomy sparing the pectoralis major muscle, and Madden group included those who had both pectoral muscles spared.

Of the 430 randomized patients, 426 remained available for our analysis. Four women were excluded since they had an indication for breast-conserving therapy and had inadvertently been included and randomized in this study. The Patey group (sparing the pectoralis major muscle) comprised 225 patients and the Madden group (sparing both pectoral muscles) comprised 201 women, as shown in Figure 1.

Thirteen different surgeons from both institutions performed either of the two procedures, the modified radical mastectomy sparing the pectoralis major muscle (Patey) or both pectoral muscles (Madden), as described previously.^6,7^

### Variables

The variables were defined as: the clinical staging using the system of the Union Internationale Contre le Cancer (UICC TNM system); duration of the operation, considered as the time elapsed between the first incision and the last suture stitch, measured in minutes; injury to the axillary vascular bundle, i.e. inadvertent sectioning of the axillary vein or artery during surgery; sectioning of nerve bundles, i.e. sectioning of the thoracodorsal nerve bundle and/or the long thoracic nerve; pneumothorax, i.e. the presence of air between the thoracic wall and the pleural space, created at the time the mastectomy was performed; wound dehiscence, i.e. loss of cohesion between the borders of the surgical wound, even when surgical stitches were used; necrosis of the skin flap, i.e. loss of skin vitality in the surgical wound, caused by inadequate blood supply to the flap; infection, i.e. the presence of inflammatory reactions, such as the presence of abscess, pain, redness, heat or edema; and hematoma, i.e. the collection of blood between the thoracic wall and the skin, during the immediate postoperative period.

The degree of difficulty found by the surgeon, during axillary dissection, was graded as follows. Technically simple procedure: easy procedure with good axillary clearance and no intervening events; moderately difficult procedure: a technically difficult procedure, in which good axillary clearance was achieved, but the surgeon experienced difficulty during the operation; technically difficult procedure: the surgeon experienced a great deal of technical difficulty and had to deal with complications, or was unable to achieve adequate axillary clearance.

For the purpose of this analysis, both surgical procedures were taken into consideration and compared with regard to the different variables, as listed above. The chi-squared and Student t tests were used, when applicable. The significance level was set at 95% (p < 0.05).

## RESULTS

The patient distribution by age, clinical stage and ancillary therapies was similar in the two groups, and as such, they were comparable. The mean age was 51.3 (± 11.7) years for the patients in the Patey group and 53.7 (± 11.3) years for the Madden group. Most of the patients had been diagnosed in clinical stage II of the disease (45.3%) (Table 1). The mean duration of the operation was 105 (± 29) and 102 (± 33) minutes (p = 0.6) for the Patey and Madden groups, respectively. The duration of hospitalization in days was 2.33 (± 0.9) for the Patey procedure and 2.29 (± 0.8) for the Madden procedure (p = 0.62). A mean of 20.3 (± 7.6) lymph nodes were resected during the Patey mastectomy and 19.8 (± 8.1) in the Madden (p = 0.52), as can be seen in Table 2.

**Table 1.** Characteristics of the women operated, according to surgical technique utilized for mastectomy: Patey^6^ or Madden^7^

	Patey n = 225	Madden n = 201	P
**Mean age (years)**	51.3 (SD ± 11.7)	53.7 (SD ± 11.3)	0.09
**Clinical staging**	**n (%)**	**n (%)**	0.1
I	35 (15.5)	22 (10.9)	
II	97 (43.1)	96 (47.8)	
III	85 (37.8)	81 (40.3)	
IV	8 (3.5)	2 (0.9)	
**Neoadjuvant chemotherapy**	**n (%)**	**n (%)**	0.8
AC	148 (65.7)	134 (66.7)	
**Adjuvant chemotherapy**	**n (%)**	**n (%)**	0.5
CMF	61 (27.1)	63 (31.3)	
FAC	86 (38.2)	82 (40.8)	
AC	14 (6.2)	8 (3.9)	
FACV	10 (0.4)	5 (2.5)	
GP	3 (1.3)	2 (0.9)	
**Endocrine therapy**	**n (%)**	**n (%)**	0.3
Tamoxifen	78 (34.7)	78 (38.8)	
**Radiotherapy**	97 (43.1)	80 (39.8)	0.5

*SD = standard deviation; AC = adriamycin and cyclophosphamide; CMF = cyclophosphamide, methotrexate and fluorouracil; FAC = fluorouracil, adriamycin and cyclophosphamide; FACV = fluorouracil, adriamycin, cyclophosphamide and vincristine; GP = gemcitabine and cisplatin.*

**Table 2.** Comparison of hospitalization and surgical characteristics, according to two different mastectomy techniques: Patey^6^ and Madden^7^

Groups	Patey n = 225	Madden n = 201	
	mean (SD	mean (SD)	p
Length of operation (minutes)	105 (± 29)	102 (± 33)	0.60
Length of hospitalization (days)	2.3 (± 0.9)	2.3 (± 0.8)	0.62
Resected lymph nodes	20.3 (± 7.6)	19.8 (± 8.1)	0.52
Involved lymph nodes	5.2 (± 7.5)	4.3 (± 6.9)	0.20

*SD = standard deviation.*

In the Patey group, Bell's nerve was sectioned in one case, while in the Madden group, there was one case of injury to the thoracodorsal nerve (p = 0.37). In the Madden group, two vascular sections were recorded: in one case the thoracodorsal artery was injured and, in the other, the axillary vein was partially sectioned, while in the Patey group, only one case of injury to the thoracodorsal artery was noted (p = 0.57). During the surgical procedures, pneumothorax was not recorded as one of the complications.

With regard to immediate postoperative complications, three patients in the Patey group and eight in the Madden group had hematomas (p = 0.16). Of the 225 patients allocated to the Patey group, 11 suffered from postoperative infection, 10 presented dehiscence of the suture line and three had flap necrosis. Of the 201 patients in the Madden group, 10 patients developed an infection, nine presented wound dehiscence and six had flap necrosis (Table 3).

**Table 3.** Analysis of intra and postoperative complications of mastectomies according to the surgical technique used: Patey^6^ and Madden^7^

Groups	Patey n = 225	Madden n = 201	
**Complications**	**n (%)**	**n (%)**	**p**
Vascular section	1 (0.4)	2 (0.9)	0.49
Nerve section	1 (0.4)	1 (0.4)	1.00
Infection	11 (4.9)	10 (4.9)	0.85
Suture line dehiscence	10 (4.4)	9 (4.5)	0.82
Necrosis	3 (1.3)	6 (2.9)	0.40
Hematoma	3 (1.3)	8 (3.9)	0.16

Concerning the degree of difficulty, 67% of the surgeons in both groups reported that the operation was a technically simple procedure. The remaining 33% from the Patey group found that the procedure presented a moderate degree of difficulty, while for the Madden group, 31% of the surgeons reported moderate difficulty, and only 2% of those who used the Madden technique reported that it was a technically difficult procedure. Thus, regarding the degree of difficulty during the operation, no statistical difference was found between the two groups.

## DISCUSSION

Since the time when Patey and Dyson^6^ and Madden^7^ described their techniques for performing modified radical mastectomies, in the 1940s and 1960s, the choice of technique for the treatment of breast cancer has been left to the surgeon. Literature searches show that no randomized studies have been carried out with the aim of identifying which one would be more beneficial or would result in fewer complications for such patients.

Some studies have been carried out to establish whether sparing the pectoralis minor muscle would bring any benefit to the patient. Argonz et al. (1985) used retrospective analysis to study a group of 70 patients who had undergone modified radical mastectomy. These patients were divided into two groups. For the first 50 patients, the surgeons used the Patey technique, while for the remaining 20, they used the Madden technique. The results for the two groups were similar.^8^

Silveira et al. (1989) analyzed the Madden technique on its own, and were able to demonstrate that the complication rates are low and that it is comparable to the Patey technique in terms of lymph node removal.^9^

Dasgupta et al. (1999) analyzed 115 mastectomy cases in which the Madden technique was used and came to the conclusion that the risk of lymphedema and the length of the surgery had been minimized. Moreover, the possibility of causing vascular or nerve injury was smaller.^10^ In spite of the lack of randomized studies in the literature that might demonstrate the superiority of one procedure relative to the other, most authors agree that the Madden technique brings about better aesthetic results and causes less morphological and functional damage to the upper limb.^8,10^

In the present study, we tried to identify the main intra and postoperative complications, in addition to factors such as the difficulty of the procedure, as reported by the surgeon, and the duration of hospitalization. It was possible to verify that, among the elements studied, the degree of difficulty of the procedure and duration of hospitalization were similar in the two groups. The intraoperative complications encountered were equivalent for the two groups, and the most commonly found complication was infection of the surgical wound. It is worth mentioning that all the patients were given a prophylactic course of antibiotics before the procedure. However, no statistical difference was found in the distribution of this complication, between the two groups.

Today, new techniques are being used to decrease the complications from mastectomies and axillary dissection.^11,12^ Lumachi et al. (2004) showed that the use of ultrasound scissors significantly reduced the total amount of serous fluid drainage from the operation site and may shorten hospital stay.^11^ In another study, Jain et al. (2004) analyzed 119 patients undergoing surgery for breast cancer and evaluated the effect of drains and fibrin sealant on the incidence of seroma formation. This study showed that drains did not prevent seroma formation, and were associated with longer postoperative hospital stay and higher pain scores after surgery for breast cancer. The use of fibrin sealant reduced the rate of seroma formation.^12^

In a recent study, a group in Canada developed evidence-based recommendations through showing that axillary dissection at levels I and II provided the same overall and disease-free survival with less morbidity, in relation to full dissection at the three levels. They therefore considered that this should be a standard practice for stage I and II breast cancer patients.^13^

Sentinel lymph node biopsy is one of the most important advances in breast cancer treatment. By using this technique, it is possible to avoid the complications related to axillary lymph node dissection for the majority of breast carcinoma patients.^14^ However, axillary clearance can only be avoided when the sentinel node is free of metastases. In order to increase the number of women for whom non-axillary dissection would be suitable even in the presence of a positive sentinel node, Schrenk et al. carried out a study with the aim of identifying a subgroup of patients with a micrometastatic sentinel lymph node and negligible risk of positive non-sentinel lymph nodes, for whom axillary lymph node dissection could be avoided. The study showed that sentinel lymph node micrometastasis of less than 0.5 mm in diameter combined with absence of lymph vascular invasion was associated with low risk of non-sentinel lymph node involvement. These authors concluded that, in such circumstances, it would be possible to avoid axillary clearance, but that prospective randomized studies would answer this question better.^15^

Today, since there is no difference in survival or distant recurrence, the choice between breast-conserving therapy with axillary dissection and modified radical mastectomy should depend on patient preference when appropriate.^13^ A less invasive method of axillary evaluation is very appealing, given the potential morbidity associated with axillary dissection. Nonetheless, a considerable number of patients will continue to have to undergo modified radical mastectomy for many years to come. Therefore, the attending surgeon needs to provide such patients with the best surgical technique possible, with the lowest complication rate. With regard to resection of the pectoralis minor, the surgeon can choose the technique according to the difficulties that may be faced during the operation, or even according to personal preference.

## CONCLUSION

This randomized trial has shown that the Patey and Madden techniques for modified radical mastectomies were similar with regard to all the criteria analyzed. The patients in the present study continue to be followed up regularly, and a report on late complications, local/regional recurrence rates, disease-free survival and overall survival rate will be released on a future date. With regard to the immediate complications and the number of resected lymph nodes, we were able to demonstrate that the techniques that spare only the pectoralis major muscle or both pectoralis muscles are equivalent and can be performed at the surgeon's discretion.
